# Assessment of the Influence on Spontaneous Pregnancy of Hysterosalpingo-Contrast Sonography

**DOI:** 10.1155/2018/4901281

**Published:** 2018-09-20

**Authors:** Gao Chunyan, Peng Bin, Yan Ping, Zhang Yue, Xiaoqing Yang, Tian Hongju, Sufen Li, Xiong Xi

**Affiliations:** ^1^Department of Obstetrics and Gynecology of the Second Clinical Medical College of Army Medical University, No. 183 Xinqiao Road, Shapingba District, Chongqing, 400037, China; ^2^Department of Medical Statistics, School of Public Health, Chongqing Medical University, No. 1 Yixueyuan Road, Yuzhong District, Chongqing 400016, China; ^3^Department of Obstetrics and Gynecology, The Second Affiliated Hospital, Chongqing Medical University, No. 74 Linjiang Road, Yu-zhong District, Chongqing, 400010, China

## Abstract

**Objective:**

Our objective was to explore whether the pregnancy rate (PR) was higher than usual after hysterosalpingo-contrast sonography (HyCoSy).

**Methods:**

We conducted a prospective observational study of 1,008 infertility patients, all of whom were examined by HyCoSy. The expected time for spontaneous pregnancy was at least 180 days after the HyCoSy exams. There were three types of HyCoSy results: type I, defined as both fallopian tubes patent; type II, defined as one fallopian tube patent with obstruction in the other; and type III, defined as both fallopian tubes obstructed. During the HyCoSy examinations, we recorded the mobility of the ovaries, injective resistance, and contrast agent venous intravasation. Before the examinations, we recorded each patient's medical history, including maternal age, infertility type, median duration of menstrual cycle, dysmenorrhea, and parity number.

**Results:**

The PR was 19.44% within 180 days after HyCoSy and it was significantly higher in the first 30 days (6.35%) (*P *<.01). The PR of type I was highest, with a rate of 32.01%, followed by the PR of type II (25.51%) and type III (15.04%) (*P *<.01). Univariate analysis showed that younger age, patency of both fallopian tubes, good ovarian mobility, and absence of injective resistance were positively related to the initiation of pregnancy (*P *<.01). Infertility type, median duration of menstrual cycle, dysmenorrhea, parity number, contrast agent venous intravasation, and identity of the sonographer were unrelated to pregnancy (*P* >.05). However, multivariate analysis showed that patency of both fallopian tubes and the absence of injective resistance were independently associated with pregnancy.

**Conclusion:**

Some infertility patients conceived successfully and naturally not long after HyCoSy, most often in the first month after the examination. Multivariate analysis showed that patency of both fallopian tubes and the absence of injective resistance were independently factors associated with the ability to conceive after HyCoSy examination.

## 1. Introduction

In China, approximately 7–10% of couples of childbearing age are confronted with infertility, which is clinically defined as the failure to achieve a pregnancy after one year or more of regular unprotected sexual intercourse [1]. In recent years, the incidence of fallopian tubal sterility has increased and become one of the most common factors leading to infertility [2]. At present, the most common methods for investigating tubal patency are laparoscopy and dye laparoscopic chromopertubation, X-ray hysterosalpingography (HSG), and hysterosalpingo-contrast sonography (HyCoSy) [3, 4]. Although HSG is the conventional modality and its diagnostic accuracy has been reported at 83.8–90.5%, its clinical use is limited by the risk of a potential iodine allergy as well as radiation exposure [5–9]. Laparoscopic chromopertubation, although considered the reference standard for the diagnosis of tubal patency, is invasive and limited by its cost and the requirement of hospitalization; therefore, it is not the preferred approach in clinical practice [10–12]. According to the literature, HyCoSy is preferred to assess the patency of tubal fimbriae; it has the advantages of accuracy, noninvasiveness, and a good safety profile [13, 14]. In addition, it does not require hospitalization, radiation exposure, or anesthesia or involve the use of iodinated contrast media. All in all, it is a simple, well-tolerated outpatient procedure that can be effectively adopted during the diagnostic workup of an infertile woman [4]. Our follow-up data show that some patients achieved natural pregnancies within several months after HyCoSy exam and that the conception occurred shortly after the HyCoSy exam. This suggested that HyCoSy might function as a therapeutic tool to enhance the chance of spontaneous conception in subfertile couples; therefore, it inspired us to initiate the present study.

Few studies have suggested that HyCoSy could directly increase pregnancy rates in the months after HyCoSy [15], although some have shown a fertility-enhancing effect and a meta-analysis showed higher rates of ongoing pregnancy after HyCoSy [13, 16]. Most of the previous studies have focused on the relationship between pregnancy rate (PR) and medical history; few trials have assessed the relationship between pregnancy and ultrasound factors. We know that the diagnostic results of HyCoSy may differ, but we do not know whether they affect the initiation of pregnancy. Therefore, the objective of this study was to describe the effect of HyCoSy on PR correlated with time elapsed as well as ultrasound factors and medical history, including maternal age, infertility type, median duration of menstrual cycle, dysmenorrhea, parity, the results of HyCoSy, mobility of the ovaries, injective resistance, contrast agent venous intravasation, and identity of the sonographer.

## 2. Materials and Methods

### 2.1. Patients

The study was conducted from July 2015 to July 2017. A total of 1,008 women with infertility were examined by HyCoSy at the Department of Obstetrics and Gynecology of the Second Clinical Medical College of Army Medical University (Chongqing, China). A complete medical history was obtained and each participant received a clinical medical examination. Patients were given appropriate information about the study objectives, in particular to establish a reason for their infertility. The expected time for spontaneous pregnancy was at least 180 days after the HyCoSy examination. The patients were divided in two groups: group A: spontaneous conception after HyCoSy, Group B: no conception after HyCoSy. The study was approved by the Ethics Committee of the Second Clinical Medical College of Army Medical University.

### 2.2. Performance of HyCoSy

A Voluson E8 ultrasound system with coded contrast imaging (GE Healthcare, Milwaukee, WI) was used. The mechanical index of the instrument was set at 0.12–0.18. An RIC5-9-D transvaginal volume transducer with a frequency range of 5.0–9.0 MHz and scanning angles from 0 to 179° was used in the examinations.

An SF6 microbubble agent (SonoVue, Bracco S.p.A., Milan, Italy) was used to prepare the contrast medium for four-dimensional (4D) HyCoSy. Specifically, the content in one SonoVue vial was first suspended with 5 mL of 0.9% normal saline, followed by further dilution of 2.5 mL with up to 20 mL of 0.9% normal saline using a 20 mL syringe. The diluted solution of SonoVue was used in the HyCoSy examination.

The 4D HyCoSy was conducted within 3–7 days after menstruation. Before the start of the procedure, we checked the mobility of both of the ovaries following a previously published method [17]. The examinations and evaluation reports were performed by five sonographers, each of whom had more than 5 years of experience in using ultrasonography to perform gynecologic and obstetric examinations.

### 2.3. Criteria for the Patency of Fallopian Fimbriae Evaluated by 4D HyCoSy

**Figure 1:**
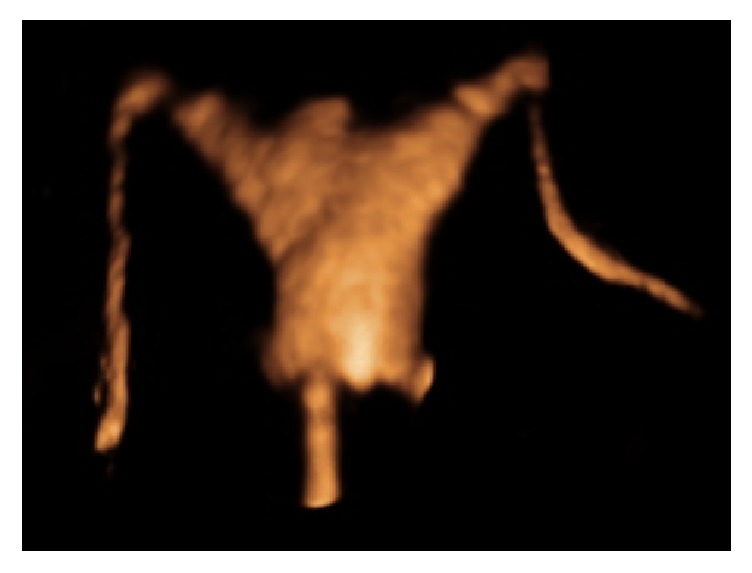
Patent fallopian tubes: both tubes developed completely and naturally.

A patent fallopian tube fills with contrast medium completely and naturally. On HyCoSy examination, there is a sheet- or ejection-like overflow of contrast medium from the fimbriae. Circular wrapping (contrast medium encircling more than half of the brim of the ovary) or semicircular wrapping (contrast medium encircling less than half of the brim of the ovary) of the contrast medium could be observed. The contrast medium diffused evenly throughout the pelvic cavity (Figure 1).

**Figure 2:**
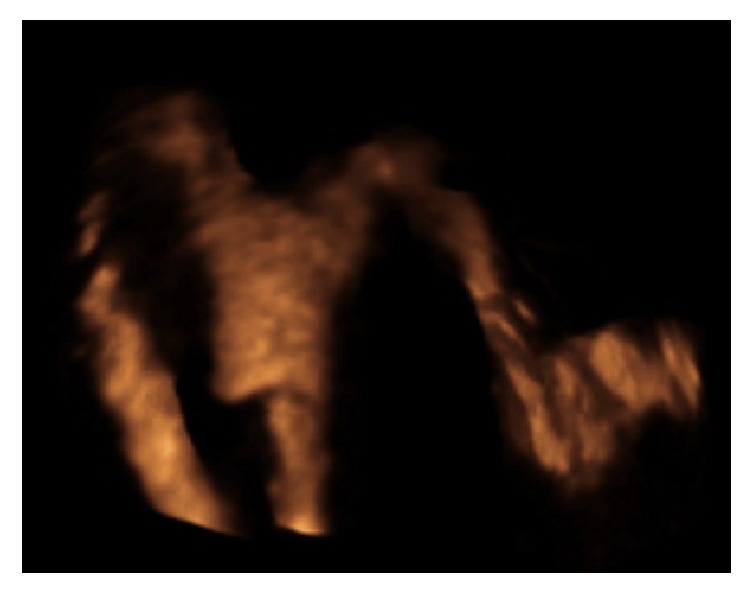
Obstructed fallopian tube: the distal segments of both fallopian tubes were markedly enlarged.

In an obstructed fallopian tube, apparent resistance is encountered during the injection of the contrast medium. The distal segment of the fallopian tube is seen to be markedly enlarged and distorted, or the fallopian tube fails to fill completely. No overflow of contrast medium from the fimbriae is observed and the contrast medium does not diffuse into the pelvic cavity (Figure 2) [18].

There were three types of HyCoSy results: type I, defined as both fallopian tubes patent; type II, defined as one fallopian tube patent with obstruction in the other; and type III, defined as both fallopian tubes obstructed.

We recorded the mobility of the ovaries, injective resistance, and contrast agent venous intravasation during the HyCoSy examinations.

The mobility of the ovaries can be accurately evaluated with a transvaginal transducer, which gently pushes and moves ovaries, to determine the precise origin of the lesion and evaluate “sliding” or “split” signs [19, 20]. Using real-time dynamic transvaginal sonography to assess the sliding sign, the sonography clinician puts the vaginal probe into posterior vaginal fornix with his right hand and gently presses the position of the ovaries with his left hand on his abdomen. Good mobility of ovaries is defined as the ovaries slipping freely, while poor mobility of ovaries is defined as immobility of ovaries.

The procedure of injecting contrast medium was conducted by an assistant who could judge the resistance of the flow.

Criteria for contrast agent venous intravasation [21–24]: contrast agent intravasation during HyCoSy may occur in the uterine myometrium or venous plexus, or both. Intravasation in the uterine myometrium can be observed as spotlike high signal enhancement from the contrast agent in a limited region, or cloudlike diffused signal enhancement within the myometrium on HyCoSy images. As for the venous plexus, there is cord or netlike signal enhancement around the uterus extending to surrounding tissue without specific direction. All the venous plexus intravasation was considered contrast agent venous intravasation.

### 2.4. Statistical Analysis

Statistical analysis was done with SPSS version 23.0 software (IBM Corporation, Armonk, NY). The chi-square test was used to evaluate categorical variables and logistic regression analysis served to identify variables correlated with spontaneous pregnancy. Kaplan–Meier curves were constructed and analyzed using the log-rank test to compare time to pregnancy in the three groups.

A value of* P *< .05 was regarded as being statistically significant.

## 3. Results

### 3.1. Patient Characteristics and Conception Time after HyCoSy

**Figure 3:**
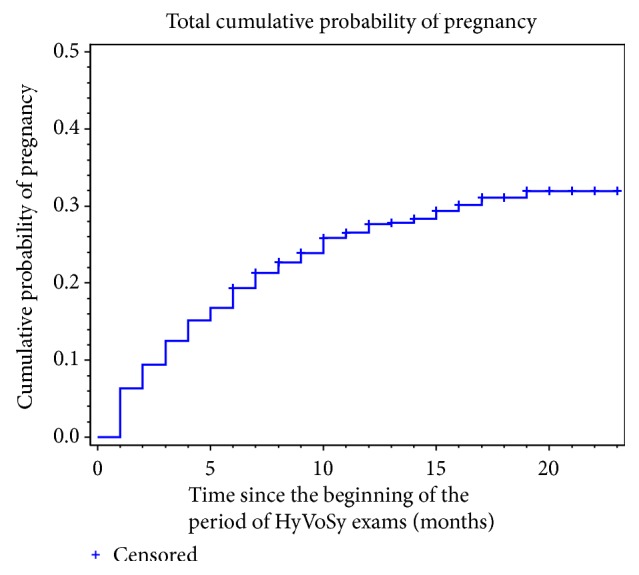
Cumulative probability of pregnancy after HyCoSy exam (months). The pregnancy rate (PR) PR was significantly higher in the first 30 days (6.35%) compared with that of the other months of observation (*P *<.01). The cumulative probability of pregnancy was 28.17% during 19 months.

The study included 1,008 female patients; of these women, 284 had gotten pregnant naturally (group A) and 724 were not pregnant (group B). The mean of “conception time” was 5.3 months. The PR was significantly higher in the first 30 days (6.35%) compared with that of the other months of observation (*P *<0.01). The cumulative pregnancy rate after HyCoSy was 19.44% within 6 months, which was also significantly higher than that of the other months of observation (*P *<.01) (Table 1, Figure 3). The cumulative probability of pregnancy was 28.17% within 19 months.

In group A, 32 women (11.27%) had their pregnancies terminated via early spontaneous abortions (within the sixth week of gestation); 9 (3.12%) had an ectopic pregnancy; and 5 (1.76%) had an induced early-stage abortion.

The ages of the 1,008 patients ranged from 18 to 47 years, with a mean of 29.25 ± 5.25 years. Of these, 25.69% of the women between the ages of 18 and 35 years conceived, as did 2.48% of women above 35 years of age. Compared with the women above 35 years of age, the PR was markedly higher in women aged 18–35 years (*P *<0.01) (Table 2).

**Figure 4:**
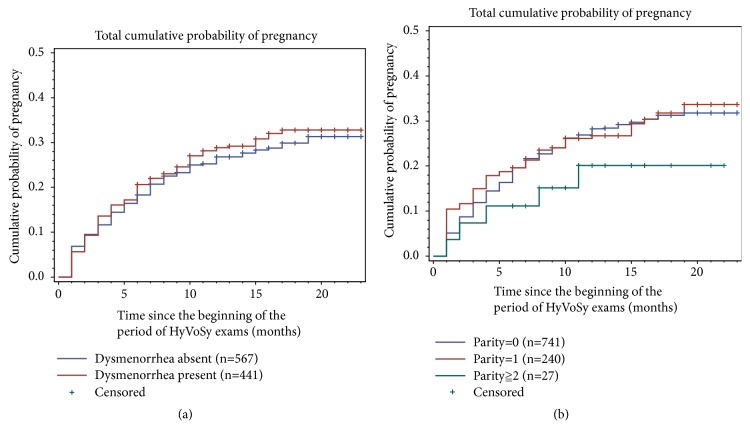
There was no difference in dysmenorrhea and parity number between pregnancy and nonpregnancy (*P *>0.05).

The proportion of woman with primary infertility was 45.63% (460 of 1,008), whereas that of secondary infertility was 54.37% (548 of 1,008). Of the 284 women who conceived, 34.15% had had primary infertility and had 65.85% secondary infertility; there was no significant difference between the two infertility types (*P *>.05). Moreover, there were also no differences between group A and group B in median duration of menstrual cycle, dysmenorrhea, and parity (*P *>.05) (Table 2, Figure 4).

### 3.2. Pregnancy Rates following Differing HyCoSy Results

**Figure 5:**
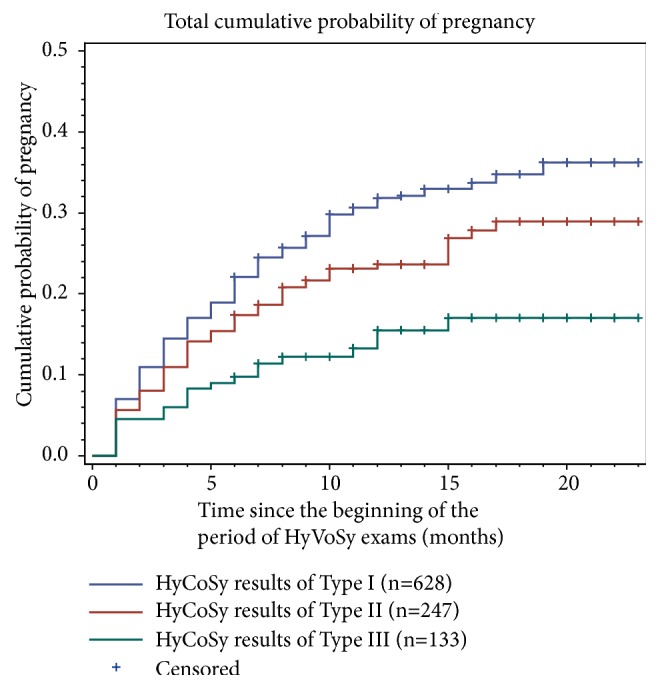
The pregnancy rate was highest in type I (32.01%), followed by type II (25.51%) and type III (15.04%) (*P *<.01).

There were three types of HyCoSy results, and the pregnancy rate was highest in type I (32.01%), followed by type II (25.51%) and type III (15.04%) (*P *< .01) (Figure 5).

**Figure 6:**
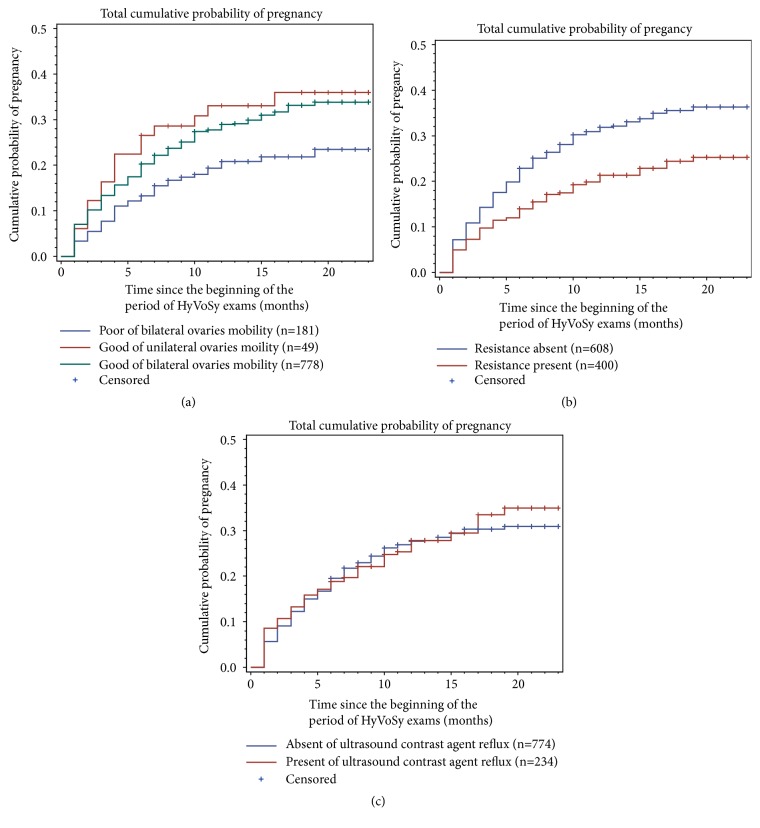
The pregnancy rate (PR) was higher in good ovarian mobility than in poor ovarian mobility (*P *<.05). The PR of the resistance absent group was 32.40%, which was markedly higher than that of the resistance present group, which was 21.75% (*P *<.01). There was no statistically significant difference between the contrast agent venous intravasation present and contrast agent venous intravasation absent groups (*P *>.05).

Ultrasound factors were also evaluated in all of the patients. The PR of women with good mobility of both ovaries was 29.43%; that of women with good mobility of only one ovary was 34.69%; and that of women with poor mobility of both ovaries was 20.09%. The PR was higher in the good mobility than poor mobility (*P *<.05). In regard to injective resistance and contrast agent venous intravasation, the PR of the resistance absent group was 32.40%, which was markedly higher than of the resistant present group, which was 21.75% (*P *<.01). There was no statistical significance between the group of contrast agent venous intravasation present (PR was 28.04%) and the group of contrast agent venous intravasation absent (PR was 28.63%) (*P *>.05) (Figure 6). There was no significant difference in the diagnoses of the five experienced sonographers (*P *>.05).

### 3.3. Clinical and Ultrasound Factors Related to PR

Univariate analysis showed that the factors related to pregnancy were ages between 18 and 35 years, both fallopian tubes patent, good ovarian mobility, and no injective resistance. Factors unrelated to pregnancy were type of infertility, median duration of menstrual cycle, dysmenorrhea, parity, contrast agent venous intravasation, and identity of the sonographer. Although multivariate analysis showed patent of fallopian tubes and no injective resistance as significant factors related to pregnancy, the younger women and good mobility of the ovaries were unrelated to pregnancy after HyCoSy exam.

## 4. Discussion

Female infertility is a common problem and tubal obstruction is its main cause. Our results show a PR of 19.35% within 6 months after HyCoSy and a significantly higher PR in the first 30 days. These results are similar to those of some other reports [15]. Our study could meet the hypothesis that the passage of liquid breaks up minor adhesions within the tubes, leading to a physiologic mechanism that enhances the possibility of a spontaneous pregnancy; however, other studies have shown that an enhanced pregnancy rate after HyCoSy could not be confirmed [18]. These discrepancies may stem from the fact that the later research focused on the contrast medium, whereas the earlier research focused on the mechanical effects of HyCoSy. In our opinion, because of the cavitation effected by the microbubble, it might produce more pressure and thus enhance recanalization of the tubes. In terms of the time to conception after HyCoSy exam, the chance of pregnancy may be improved shortly after the examination because the PR declined gradually as time went on.

Although some studies on pregnancy and HyCoSy have been reported [25] and most of this research has involved the relationship between the PR and medical history, there is no research on PR and ultrasound factors. This study found that type I (with both fallopian tubes patent) had the highest PR, followed by type II (with only one fallopian tube patent), in which the PR was lower. Lastly, there was type III, with both fallopian tubes obstructed. This suggested that the results of HyCoSy were generally reliable and could reflect the function of the fallopian tubes accurately; the result was similar with some researches stating that HyCoSy may significantly reduce the need for laparoscopy as a reference standard [26]. The results also suggested that the women with patent tubes had a higher PR; however, we must admit that some cases of obstruction were misdiagnosed. Since it is difficult to achieve pregnancy with obstructed fallopian tubes, there may be some false-positives in our results. This conclusion is consistent with some other research in which it was determined that it is more difficult to diagnose tubal occlusion than tubal patency, which can lead to some false-positive results [8].

In addition to analyzing the results of HyCoSy related to pregnancy, we analyzed some ultrasound factors that might influence the accuracy of HyCoSy indirectly, including mobility of the ovaries, injective resistance, contrast agent venous intravasation, and identity of the sonographer. It had been reported that ovarian mobility is a sonographic “soft marker” in evaluating deep infiltrating endometriosis and extensive pelvic adhesions, both of which are associated with subfertility [27, 28]. Furthermore, some research has indicated that injective resistance and contrast agent venous intravasation are associated with diagnostic results [8, 21–30]; however, which of these is most relevant to pregnancy has not been reported. Our univariate analysis showed that patency of the tubes, mobility of the ovaries, and injective resistance are related to pregnancy after HyCoSy exam and that contrast agent venous intravasation and the identity of the sonographer are not related to pregnancy after HyCoSy exam, whereas multivariate analysis showed that patent fallopian tubes and no injective resistance were the related factors. Injective resistance had a high degree of consistency with the diagnostic results of HyCoSy because injective resistance was a relatively objective factor judged by an assistant during the process of HyCoSy. Mobility of the ovaries was a relatively subjective factor judged by the sonographers before HyCoSy; therefore, this was not a factor associated with pregnancy after HyCoSy.

The main limitation to this study is that it was not case-controlled, although our sample size was large. We had an impression that the unique cavitation effect of the contrast agent might have had a positive effect on tubal patency, but our study involved only a self-control observation and thus could not prove our assumption. Therefore, a large-scale prospective randomized study should be planned to prove the hypothesis.

In conclusion, we found that some previously infertile women were able to conceive successfully and naturally after HyCoSy and that the rate of such conceptions was significantly higher in the first 30 days. Furthermore, multivariate analysis showed that patency of both fallopian tubes and absence of injective resistance were the independent factors correlating with pregnancy.

## Figures and Tables

**Table 1:** Cumulative probability of pregnancy after HyCoSy exams (months).

Month(s)	Number of pregnancies	Cumulative number of pregnancies	Cumulative probability of pregnancy (%)
1	64	64	6.45
2	31	95	9.52
3	31	126	12.60
4	27	153	15.28
5	16	169	16.87
6	26	195	19.44
7	19	214	21.33
8	13	227	22.62
9	10	237	23.61
10	17	254	25.30
11	5	259	25.79
12	8	267	26.59
13	1	268	26.69
14	3	271	26.98
15	5	276	27.48
16	3	279	27.78
17	3	282	28.08
18	0	282	28.08
19	2	284	28.17

**Table 2:** General characteristics of the two groups.

Characteristic	Group A (n=284)	Group B ( n=724)	*P* Value
Age			
Median (years)	28.48 ± 4.80	29.56 ± 5.39	0.013
Age group—no./total no. (%)			
18–35 years	259 (25.69)	604 (59.93)	0.002
> 35 years	25 (2.48)	120 (11.90)	
Primary infertility—no. (%)	97 (34.15)	263 (36.33)	0.518
Secondary infertility	187 (65.85)	461 (63.67)	
Median duration of menstrual cycle—days	30.88 ± 8.67	31.94 ± 12.63	0.976
Dysmenorrhea—no. (%)	567 (72.66)	441 (70.75)	0.503
Parity number			
Nulliparity	211 (74.29)	530 (73.20)	0.651
One live birth	68 (23.94)	172 (23.76)	
Two live births	5 (1.76)	22 (3.04)	

## Data Availability

The data used to support the findings of this study are available from the corresponding author upon request.
